# Characterisation of Structural Proteins from Chronic Bee Paralysis Virus (CBPV) Using Mass Spectrometry

**DOI:** 10.3390/v7062774

**Published:** 2015-06-23

**Authors:** Aurore Chevin, Bruno Coutard, Philippe Blanchard, Anne-Sophie Dabert-Gay, Magali Ribière-Chabert, Richard Thiéry

**Affiliations:** 1 ANSES, Sophia-Antipolis Laboratory, Bee Diseases Unit, BP 111, 06902 Sophia Antipolis, France; E-Mails: aurore.chevin@free.fr (A.C.); philippe.blanchard@anses.fr (P.B.); magali.chabert@anses.fr (M.R.-C.); 2 Aix-Marseille Université, CNRS, AFMB UMR 7257, 13288 Marseille, France; E-Mail: bruno.coutard@afmb.univ-mrs.fr; 3 Institute of Molecular and Cellular Pharmacology, IPMC, UMR6097 CNRS, 660 route des Lucioles, 06560 Valbonne, France; E-Mail: gay@ipmc.cnrs.fr

**Keywords:** chronic bee paralysis virus (CBPV), nano-HPLC, MALDI-TOF/TOF, protein identification, hypothetical structural protein (hSP), predicted structural protein (pSP), anti-pSP antibodies

## Abstract

Chronic bee paralysis virus (CBPV) is the etiological agent of chronic paralysis, an infectious and contagious disease in adult honeybees. CBPV is a positive single-stranded RNA virus which contains two major viral RNA fragments. RNA 1 (3674 nt) and RNA 2 (2305 nt) encode three and four putative open reading frames (ORFs), respectively. RNA 1 is thought to encode the viral RNA-dependent RNA polymerase (RdRp) since the amino acid sequence derived from ORF 3 shares similarities with the RdRP of families *Nodaviridae* and *Tombusviridae*. The genomic organization of CBPV and *in silico* analyses have suggested that RNA 1 encodes non-structural proteins, while RNA 2 encodes structural proteins, which are probably encoded by ORFs 2 and 3. In this study, purified CBPV particles were used to characterize virion proteins by mass spectrometry. Several polypeptides corresponding to proteins encoded by ORF 2 and 3 on RNA 2 were detected. Their role in the formation of the viral capsid is discussed.

## 1. Introduction

**Figure 1:**
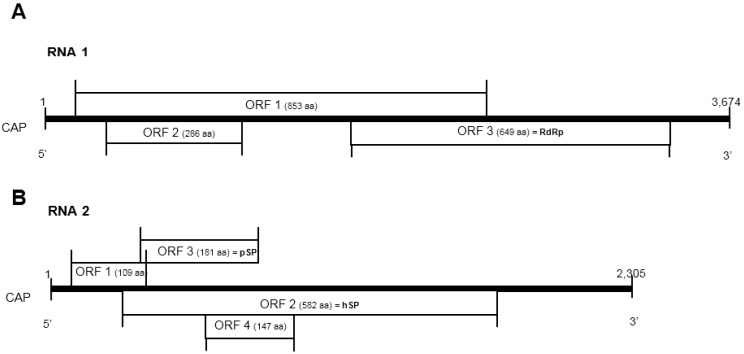
Diagram of the predicted genome organization of CBPV RNA 1 (**A**) and RNA 2 (**B**). Seven putative ORFs are indicated with their positions and putative amino acid sequence length. aa: Amino acid; RdRp: RNA-dependent RNA polymerase; pSP: predicted structural protein; hSP: hypothetical structural protein.

Chronic paralysis is an infectious and contagious disease of adult honeybees (*Apis mellifera* L.). It is known to induce a cluster of clinical signs, such as trembling and flightless bees crawling at the hive entrance [1]. The viral agent that causes this disease is the chronic bee paralysis virus (CBPV). CBPV shows neurotropism in concomitance with observed clinical signs [2]. The viral particle is anisometric and non-enveloped, its size being about 30–60 nm in length and 20 nm in width [3]. CBPV is a positive single-stranded fragmented RNA virus; its genome is composed of two major RNA fragments that have been completely sequenced, RNA 1 (3674 nt) and RNA 2 (2305 nt) [4]. Bioinformatic analysis of RNA sequences has shown that there are seven putative overlapping open reading frames (ORFs), three on RNA 1 and four on RNA 2 [4] (Figure 1).

Comparing amino acid sequences deduced from each ORF with protein sequence databases, only ORF 3 on RNA 1 shares significant similarities with the RNA-dependent RNA polymerases (RdRp) of positive single-stranded RNA viruses. Indeed, this putative RdRp of CBPV possesses the eight conserved domains (I–VIII), and the specific catalytic subunit containing the GDD motif. Based on the eight conserved domains of RdRp, a phylogenetic study shows that CBPV seems to occupy an intermediate position between families *Nodaviridae* and *Tombusviridae* [4]. CBPV shares some features with these virus families [5], suggesting that the ORFs on RNA 1 encode non-structural proteins and ORFs on RNA 2 encode structural proteins. Furthermore, the amino acid sequence of ORF 2 and 3 on RNA 2 yield two proteins with predicted molecular masses of about 65.5 kDa and 19.7 kDa, respectively. These molecular masses may correspond to previously immunodetected proteins, estimated at 75 and 20 kDa [5]. The molecular mass of the 20 kDa protein is similar to the single capsid protein of about 23.5 kDa proposed by Bailey [6]. Therefore, ORF 3 on RNA 2 may encode a capsid protein, called predicted structural protein (pSP) [7]. Despite the current knowledge on CBPV, this virus cannot yet be assigned to any viral family. However, genomic sequences of two novel honeybee viruses have been identified, Lake Sinai virus 1 and 2 (LSV 1 and LSV 2). These genomes have similarities with CBPV RNA 1 and the ORFs encoding RdRp show 25% amino acid identity with CBPV. Amino acid phylogeny of RdRp of families *Nodaviridae* and *Tombusviridae* places LSV 1 and 2 on the same branch as CBPV [8]. A unique nodavirus, called Mosinovirus, was recently placed between the monopartite LSV and bipartite CBPV by phylogenetic analysis [9]. In a recent study Kuchibatla *et al*. [10] made some predictions about CBPV structure and composition: (I) ORF1 of RNA1 contains a Methyltransferase-Guanylyltransferase; (II) pSP could be membrane-associated protein with 3 or 4 transmembrane segments, and potential *N*-glycosylation sites, which is conserved in a wide range of insect and plant viruses; and (III) hSP could be a virion glycoprotein with two transmembrane segments. These predictions were made using powerful sequence similarity search methods, but at present experimental evidence are lacking to confirm these hypotheses.

Conventionally, protein characterization from virus particles requires pure viral particle preparation, protein denaturation, electrophoretic separation and identification of proteins by western blot or by amino terminal Edman sequencing. Since mass spectrometry (MS) has emerged, a combination of techniques has been developed, thereby providing new tools for structural investigations on viruses. One-dimensional (1D) electrophoresis and liquid chromatography (LC) coupled to tandem MS (MS/MS) analysis has proved to be useful for the exploration of virus proteomes such as the murine cytomegalovirus [11], *Acanthamoeba polyphaga* mimivirus [12] or *Autographa californica* multiple nucleopolyhedrovirus [13]. Contrary to proteomic research on DNA viruses, proteomic studies have been performed on a limited spectrum of RNA viruses probably because the number of proteins in RNA viruses is smaller and proteomic approaches are generally not necessary. However, two RNA viruses have been intensively analyzed: Severe acute respiratory syndrome-associated coronavirus (SARS-CoV) [14] and Human immunodeficiency virus 1 (HIV-1) [15]. Using SDS-Page followed by Electrospray ionization (ESI) MS/MS, structural proteins of SARS-CoV have been identified as components of the nucleocapsid and envelope. The LC coupled to MS/MS analysis of HIV-1 even identified cellular proteins associated with viral proteins, providing other ways to study HIV-1 assembly, infection and pathogenesis.

Although seven ORFs have been predicted from the analysis of the CBPV genome, little is known about the biological significance of these ORFs. CBPV proteins need to be characterized to gain insight into viral structure and, consequently, better classify this virus. Here, we describe the first proteomic analysis of CBPV. We examined a CBPV preparation from honeybee heads that was purified on a sucrose gradient, followed by SDS-Page separation and nano-HPLC coupled to a matrix-assisted laser desorption/ionization-time of flight/time of flight (MALDI-TOF/TOF) mass spectrometer. Our analysis revealed two CBPV proteins that are likely to be structural proteins. These new results provide important information on CBPV and they will help to better understand its capsid composition.

## 2. Materials and Methods

### 2.1. Virus Purification

Ten forager and worker honeybee (*Apis mellifera* L.) from a colony were collected to test CBPV-free by real time RT-PCR [16,17]. Emerging bees were sampled from this colony and were maintained at 30 °C in small cages supplied with sugar candy food sources and sucrose syrup complemented with protein L. After one week, 400 adult bees were inoculated via intra-thoracic injection using 10^4^ CBPV genome copies per injection. CBPV particles were purified from heads of experimentally infected bees as previously described [4]. Briefly, 400 heads were manually crushed and the homogenate underwent successive centrifugations. Then, concentrated CBPV was separated on a 10% to 40% (*w*/*v*) sucrose gradient and centrifuged for 4.5 h at 15 °C (SW41 rotor). Viral fractions were collected, centrifuged, and the pellet was resuspended in 0.01 M phosphate buffer (PB). A sample of purified CBPV particles was negatively stained to be examined under a transmission electron microscope (Centre commun de microscopie appliquée, Microscopy Facility, University of Nice-Sophia Antipolis).

The purified virus was quantified using real time RT-PCR [16,17] and the absence of other honeybee RNA viruses genomes: acute bee paralysis virus (ABPV) [18], Black queen cell virus (BQCV), deforming wing virus (DWV) [16], Israeli acute bee paralysis virus (IAPV) [19], and Sacbrood virus (SBV) [20] was checked using conventional RT-PCR. Finally, protein concentration was determined using Lowry’s method [21].

### 2.2. In-Gel Digestion for Protein Identification

The proteins from CBPV purification (50 µg) were separated on a 12% and “Any-kD” Mini-PROTEAN TGX precast gels (Bio-Rad, Hercules, CA, USA) under denaturing conditions and stained with Coomassie brilliant blue R-250 (Pierce). Protein bands or regions were manually excised from the gel and cut into pieces. The gel pieces were destained by adding 100 µL of H2O/ACN (acetonitrile) (1/1). After 10 min incubation with vortexing, the liquid was discarded. This procedure was repeated twice. Gel pieces were then rinsed (15 min) with ACN and dried under vacuum. Gel pieces were reswelled in 50 µL of 100 mM DTT (dithiothreitol) in 100 mM NH_4_HCO_3_. They were incubated for 45 min at 56 °C, and then cooled down to room temperature. The DTT solution was replaced with 100 µL of 55 mM iodoacetamide in 100 mM NH_4_HCO_3_. After 1 h incubation at room temperature, the solution was discarded and the gel pieces were washed by adding successively (1) 100 µL of H_2_O/ACN (1/1), repeated twice and (2) 100 µL of ACN, and they were dried in a vacuum. Then gel pieces were reswelled in 50 µL of 50 mM NH_4_HCO_3_ buffer containing 12.5 ng/µL trypsin (modified porcine trypsin sequence grade, Promega, Madison, WI, USA), and incubated for 1 h at 4 °C. Then the solution was removed and replaced by 50 µL of 50 mM NH_4_HCO_3_ buffer (without trypsin) and incubated for 18 h at 37 °C. After trypsin digestion, the solution was transferred to an eppendorf tube and tryptic peptides were isolated by extraction with (1) 50 µL of 1% formic acid (FA) in water (*v*/*v*) (10 min at room temperature) and (2) 50 µL ACN (10 min at room temperature). Peptide extracts were pooled, concentrated under vacuum and solubilized in 10 µL of 0.1% trifluoroacetic acid (TFA) in water (*v*/*v*).

### 2.3. Nano-HPLC MALDI-TOF/TOF Analysis (Peptide Separation and Fractionation and Mass Spectrometry)

Peptide separation was carried out using a nano-HPLC offline (DIONEX, U3000, Courtaboeuf, France) coupled with a MALDI-TOF/TOF mass spectrometer (4800 plus, Applied Biosystems, Waltham, MA, USA).

The peptide solution was concentrated on a µ-Precolumn Cartridge Acclaim PepMap 100 C18 (i.d. 5 mm, 5 µm, 100 Å, DIONEX, LC Packings) at a flow rate of 20 µL/min and using solvent containing 98%/2%/0.04% H_2_O/ACN/TFA (*v*/*v*). Then, peptide separation was performed on a 75 µm i.d. × 150 mm (3 µm, 100 Å) Acclaim PepMap 100 C18 column (DIONEX, LC Packings) at a flow rate of 200 nL/min and with detection at 214 nm. The solvent systems were: (A) 100% water, 0.05% TFA; (B) 100% ACN, 0.04% TFA. The following gradient was used t = 0 min 100% A; t = 3 min 100% A; t = 63 min, 80% B; t = 64 min, 100% B; t = 68 min 100% B (temperature was set at 30 °C).

For offline nano-HPLC-MALDI-TOF/TOF-MS and MS/MS analyses, fractions were collected on a Opti-TOF LC/MALDI target (123 mm × 81 mm, Applied Biosystems) and fractionation was done using the Probot fractionation robotor (DIONEX, LC Packings). Matrix solution (α-cyano-4-hydroxycinnamic acid, 2.5 mg/mL in 50% water, 50% ACN, 0.1% TFA solution) and nano-HPLC fractions were mixed (at a ratio of 4:1, matrix:fractions) and collected every 20 s (208 fractions were collected per run).

MALDI-TOF/TOF-MS analysis: MS spectra were recorded automatically in a mass range of 500–4000 Da resulting from 200 laser shots of constant intensity. Data were collected using a 4000 series Explorer (Applied Biosystems) allowing for an automatic selection of peptide masses for subsequent MS/MS experiments. The MS/MS spectra, each acquired using 1000 laser shots, were further processed using the 4000 series Explorer. Finally, all raw data were transferred to ProteinPilot software (Applied Biosystems, MDS Analytical Technologies) and protein identification was processed using the ParagonTM algorithm.

### 2.4. Western Blot Analysis

The proteins from the CBPV purification (25 µg) in presence or absence of β-mercaptoethanol were separated on a 12% SDS-Page gel and transferred onto a nitrocellulose membrane (Bio-Rad) via semidry electrophoresis transfer. Membranes were blocked in 5% (*w*/*v*) nonfat-dry milk in TBST (10 mM Tris-HCl pH 7.4, 150 mM NaCl, 0.05% (*w*/*v*) Tween 20) and all antibody incubations were done in the same buffer. Three different primary antibodies were used. The anti-RdRp antibodies were produced from rabbits immunized with recombinant RdRp produced in E. coli with a *N*-terminal hexa-histidine tag and purified under non-denaturing conditions following a routine protocol [22]. The anti-pSP antibodies were generated from rabbits immunized with two synthesized peptides (SRRPSRPRRSILDR, position 18–31, and AVLSSARRSEKFAY, position 103–117) (P.A.R.I.S. Antibodies, Compiègne, France). Absorbed polyclonal anti-CBPV antibodies were also used [23]. The three antibodies were used at the dilution of 1:200. Alkaline phosphatase-conjugated Affinipure goat anti-IgG of rabbit (Jackson ImmunoResearch, West Grove, PA, USA) was used as secondary antibody at dilution of 1:1000. The signal was detected with an alkaline phosphatase conjugate substrate kit (Bio-Rad).

### 2.5. Computer-Assisted Sequence Analysis

Putative amino acid sequences of pSP (GI: 188572677) and hSP (GI: 188572676) were used to generate the GRAVY score value with ProtParam [24] and hydrophobicity plots with the algorithm of Kyte and Doolittle [25] using PROTSCALE [26]. Then, secondary-structure predictions were performed using PsiPred [27]. The amino acids sequence of pSP was compared with protein sequence databases using HHpred in order to sequence similarity to known proteins [28]. Using AUG_hairpin [29], the downstream stem-loop structures of ATG codons were predicted with about 100 nucleotides starting from the ATG codons at positions 869 and 920 of RNA 2.

## 3. Results

### 3.1. Western Blot Analysis of Virions

**Figure 2:**
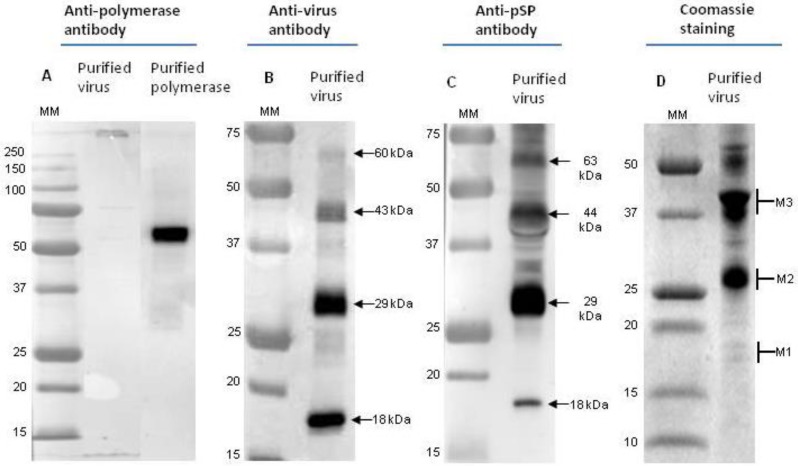
(**A**) Western blot analyses of CBPV purification, using the anti-RdRp antibodies; (**B**) using the anti-CBPV antibodies; (**C**) the anti-pSP antibodies (**D**). Coomassie blue stained of a purified CBPV sample (25 µg of proteins) on an “Any kD” gel. MM: Molecular mass in kDa.

After sucrose gradient centrifugation, the purified viral particles were checked by electron microscopy and the negatively stained purified CBPV sample showed mostly anisometric and ellipsoidal particles, as previously observed [4]. Moreover, the micrograph confirmed that the virions were morphologically intact (data not shown). The purified CBPV particles were used for western blotting with various antisera (Figure 2). Although anti-RdRp antibodies recognize the recombinant protein corresponding to the fragment of RdRp used for rabbit immunization, RdRp was not detected in the purified CBPV sample (Figure 2A). By using anti-CBPV antibodies raised against purified viral particles, four polypeptides with molecular masses of approximately 60, 43, 29 and 18 kDa were detected (Figure 2B). Surprisingly, a similar pattern was observed when using anti-pSP antibodies. Although the predicted molecular weight of pSP is 19.7 kDa, four bands were observed (63, 44, 28 and 18 kDa) (Figure 2C). We thus hypothesize that the 18 kDa polypeptide corresponds to pSP, whereas other bands could correspond to different oligomerization states of pSP (see discussion). To check whether pSP is bound by disulfide bonds and forms multimers, SDS-Page and western blotting were performed with or without β-mercaptoethanol. No significant differences were observed, suggesting that disulfides bonds are not involved in the oligomerization of pSP (not shown).

### 3.2. Detection of pSP and hSP Peptides by Mass Spectrometry

Further characterization of the proteins detected by immunoblotting was undertaken by MS/MS analysis. The purified CBPV sample was separated on a gel that offered optimal resolution for low molecular mass proteins. After Coomassie blue staining, as shown in Figure 2D, three bands corresponding to immunodetected proteins around 18, 28 and 44 kDa (M1, M2 and M3) were excised and underwent trypsin digestion and extraction as described in the Materials and Methods section. Surprisingly, the MS/MS analysis revealed that M1 (18 kDa) and M3 (44 kDa) contained peptides corresponding to pSP and hSP, whereas only pSP peptides were detected in M2 (28 kDa) (Table 1). A band around 60 Da was also analyzed but identified peptides had a confidence value lower than 95%, which is insufficient to confirm the presence of pSP and hSP.

**Table 1:** Identification of immunodetected proteins (fractions M1, M2, M3) from purified CBPV samples using nano-HPLC coupled to MALDI-TOF/TOF.

Fraction	Approximate Molecular Weight (kDa)	Viral Protein	Number of Peptides (Confidence of Identity > 95%)	Sequence of Identified Peptides
M1	18	pSP	1	FIGDFITEHPEQTIGAVAVSAAVLSSAR
		hSP	2	QIPIANFNEFLIK, LLEGPDEWLLVTAR
M2	29	pSP	4	FIGDFITEHPEQTIGAVAVSAAVLSSAR, LQTVNALK, SILDR, SIVVTLGQK
M3	44	pSP	3	FIGDFITEHPEQTIGAVAVSAAVLSSAR, LQTVNALK, SIVVTLGQK
		hSP	7	QIPIANFNEFLIK, LFHYYPPLQLR, IDTQATLSELR, LLEGPDEWLLVTAR, FFPLQPLAR, ADNPDSLLEVLPVLVSADIK, YDWLGSGGSYCLVQPDR

**Figure 3:**
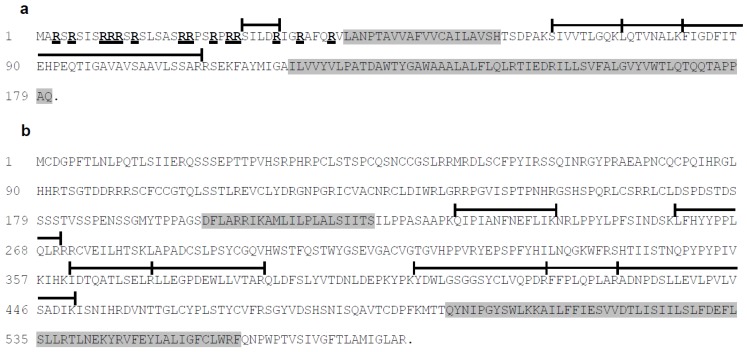
(**a**) Amino acid sequences of pSP (181 amino acids); (**b**) Amino acid sequences of hSP (582 amino acids); Bars represent the peptides identified using mass spectrometry, the percentage of coverage is 28% for pSP and 16% for hSP. Hydrophobic domains corresponding to the predicted alpha-helices are indicated in grey. The arginine residues from the N-terminal region of pSP are shown in bold and underlined. Points correspond to the stop codon.

For hSP, we detected only two peptides in M1, whereas in M3, we identified seven peptides. However, no peptides corresponding to the *N*-terminal region of hSP were detected. The localization of the identified peptides relatively to the pSP and hSP aminoacid sequences is shown in Figure 3. It is noteworthy that very few peptides have been identified for pSP and hSP, leading to low sequence coverage, 28% and 16% respectively. This low rate may be due to various properties of these proteins. For example, pSP and hSP sequences revealed two hydrophobic regions, and all identified peptides were outside of these hydrophobic regions. Additionally, pSP contains an arginine cluster leading to tryptic peptides that are too small to be analyzed.

By using mass spectrometry we did not detect any peptides derived from the ORFs of CBPV RNA 1. In addition the CBPV RdRp was not immunodetected in the purified CBPV particles by western blotting using a specific anti RdRp antibody. Therefore it is most likely that this protein is not encapsidated. In contrast to negative-stranded RNA viruses, it is currently accepted that in positive-stranded RNA viruses, the RdRp proteins are not incorporated into viral particles.

Western blottings and mass spectrometry experiments demonstrated the presence of a protein of around 18 kDa, corresponding to the predicted molecular mass of pSP (19.7 kDa). These data thus confirmed that the putative pSP protein encoded by ORF 3 of CBPV RNA 2 is a component of the virion.

Protein hSP, which is composed of 582 amino acids with a theoretical molecular mass of 65.5 kDa, seems to be a viral capsid component too. By MS/MS analysis, hSP peptides were identified on bands between 50 and 68 kDa. This protein thus seems to be present at its expected molecular weight. However, hSP peptides were also detected in lower bands at about 18 and 44 kDa and no peptide matching to the N-terminal part of the ORF was detected.

### 3.3. Analysis of the Amino Acid Sequences of pSP and hSP

The grand average hydropathicity (GRAVY) value score of proteins provide an image of the hydrophobicity of the whole protein. A positive score indicates a hydrophobic protein and a negative score indicates a hydrophilic protein. The GRAVY of pSP is 0.300 according to ProtParam. Furthermore, of its 181 residues, this protein possesses 102 aliphatic residues. The hydrophobicity plot determined two hydrophobic regions, one internal (residues 43 to 58) and a larger one at the *C*-terminus (residues 112 to 180) (Figure 3a). In the *N*-terminal region, encompassing residues 3 to 40, there are 14 arginines that confer a high density of positive charges. The region from residue 4 to residue 17 contains five arginine residues and is organized in a predicted α-helical secondary structure. Contrary to pSP, the GRAVY of hSP is −0.189 suggesting an overall hydrophilic protein. Despite this negative score, the hydrophobicity plot predicted two hydrophobic regions. Like pSP, hSP contains one internal predicted hydrophobic region (residues 200 to 222) and another one at the *C*-terminus (residues 504 to 560) (Figure 3b). In addition, the secondary structure predictions indicated that these hydrophobic regions have an α-helical organization.

**Figure 4:**
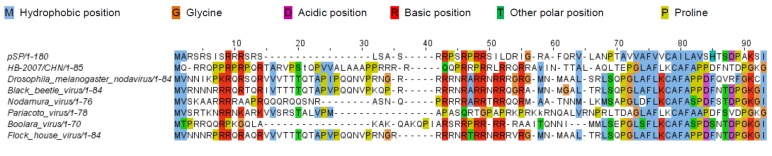
The N-terminal region of CBPV pSP and several alphanodaviruses. Similarities were detected using HHpred, edited manually and visualized using Jalview [30]. Reference numbers: pSP (GeneBank: ACO82565.1), Alphanodavirus HB-2007/CHN (GeneBank: ADF97522.1), Drosophila melanogaster American nodavirus (GeneBank: ACU32796), Black Beetle virus (SwissProt: P04329.1), Nodamura virus (SwissProt: P12871.1), Pariacoto virus (GeneBank NP_620111.1), FHV Flock house virus (SwissProt: P12870.1).

HHpred detected sequence similarity between the N-terminal region of pSP (ORF 3, RNA 2) and the N-terminal region of the capsid proteins of several alphanodaviruses (Figure 4). By contrast, no homolog of ORF 2 could be detected using this program.

## 4. Discussion

In this study, we identified for the first time distinct peptides in CBPV particles purified from honeybee heads using mass spectrometry analysis. These peptides were derived from RNA 2 ORF 2 and ORF 3, which are thought to encode two putative proteins: the hypothetical structural protein (hSP) and predicted structural protein (pSP) [8]. These ORFs are therefore most likely expressed. Interestingly, novel viral sequences highly homologous to CBPV were found in a dipteran host [31]. This new putative virus, called anopheline-associated C virus or AACV possesses homologs of RNA2 ORF2 and ORF3, but lacks homologs of RNA 2 ORF1 and ORF4.

The pattern observed when using anti-CBPV and anti-pSP antibodies were similar and in agreement with Ribière *et al.* [18].

However, using anti-pSP antibodies, the immunoblot revealed three additional pSP states. One protein was detected at an apparent mass of 28 kDa, which is about 10 kDa larger than the predicted mass. The discrepancy between the apparent and predicted molecular masses of pSP may be due post-translational modifications, raising its apparent molecular mass by about 10 kDa. Post-translational modifications of viral capsid proteins, such as phosphorylation and glycosylation, have been reported for other non-enveloped viruses [32,33,34], which may explained this discrepancy. Two other proteins were revealed at 44 and 63 kDa, suggesting that pSP may be a dimer and a trimer, or a dimer of post translationally modified protein as described earlier, e.g., for the P12 domain of the Mason-Pfizer monkey virus Gag protein able to form di, tri and tetramers resistant to SDS Page [35]. However, the immunodetection profile was the same, with or without β-mercaptoethanol, indicating no inter-chain binding by disulfide bonds. Therefore, pSP may be associated with itself or with another protein with high affinity.

The unexpected pSP and hSP peptide patterns obtained by mass spectrometry are quite puzzling. Several hypotheses may explain these results.

First, hSP may be an immature protein capsid precursor, which is further cleaved to yield two or several capsid proteins. This type of processing has been reported in the genera *Aphtovirus* [36], *Enterovirus* [37] and *Alphanodavirus* [38].

**Figure 5:**
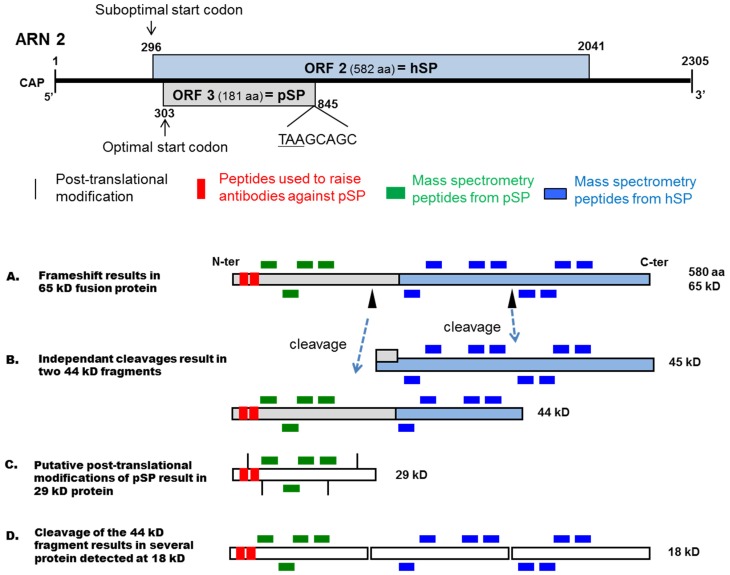
Putative translation mechanism and proteins maturation of ORFs 2 and 3 on CBPV RNA 2. Top: Diagram of predicted genome organisation of ORFs 2 and 3 on CBPV RNA 2. The ORFs are indicated with their positions and putative amino acid sequence length. aa: amino acid, pSP: predicted structural protein, hSP: hypothetical structural protein; (**A**) Putative fusion protein capsid of about 65 kD resulting from translation initiation from the first ATG of ORF 3 and recovery of ORF 2 around the STOP codon of ORF 3; (**B**) The p65 may be cleaved by means of two non-simultaneous cleavage sites (arrowheads) into two proteins of about 45 kD, pSP until mid of p65 and mid-*C*-terminal region of p65 corresponding to immunodetected protein about 44 kD; (**C**) The immunodetected protein of about 28 kD may be pSP alone with post-translational modifications; (**D**) The products of maturation of about 44 kD may be cleaved into several proteins corresponding to immunodetected proteins at about 18 kD.

Another hypothesis would involve a combination of ORF 3 and ORF 2, yielding one fusion capsid protein (p65). This protein of about 65 kDa would be composed of 580 amino acids containing the whole sequence of pSP and mid-*C*-terminal region of hSP (Figure 5A). Protein p65 would result from the initiation of the translation at the first ATG codon of ORF 3, combined with a mechanism bypassing the ORF 3 stop codon and leading to the recovery of ORF 2 frame. Although the ATG of ORF 2 was found in a Kozak context [4], it is noteworthy that ORF 3 (pSP) is overlapping the 5′ end of ORF 2 (*N*-terminal region of hSP). A novel analysis of translation initiation sites showed that ATG of ORF 2 is in a suboptimal Kozak context contrary to the ATG of ORF 3. Indeed, intitiation codons have a strong preference for the pattern A/GxxATGG, especially the G at +4 and the G at −3 [39]. The ATG of ORF 3 is in this situation (GxxATGG), contrary to the ATG of ORF 2 (TxxATGT). Thus, even if the ATG of ORF 2 is in front of the ATG of ORF 3, it is most likely that the ribosomes would rather initiate translation at the ORF 3 ATG almost all the time. Altogether, this could explain the lack of identification of peptides from the *N*-terminal region of hSP, and the presence of peptides from the mid to *C*-terminal region of hSP. Although no pseudoknot was detected in this region, the production of p65 associated to cleavages could also explain why no peptides were identified in *N*-terminal region of hSP and the unexpected apparent molecular mass of pSP and hSP. Indeed, the repartition of identified peptides from pSP and hSP could correspond to the location of these peptides on the sequence of p65 at about 65 kDa (Figure 5A). Moreover, the synthesized peptides used for the production of anti-pSP antibodies were located in the *N*-terminal region of pSP thus corresponding to the *N*-terminal region of p65 (Figure 5A). If we postulate that p65 possesses two cleavage sites (Figure 5A, arrowheads), the proteins having a molecular mass lower than 65 kDa on immunoblots would be cleavage products. If this is true, the 44 kDa band (Figure 2C) could result from the detection of two cleaved proteins: pSP until middle of p65 and mid-*C*-terminal region of p65 (Figure 5B). In this model the 29 kDa protein would be the post-translationally modified pSP (Figure 5C). For 18 kDa, the proteins would correspond to pSP and cleaved products of the two proteins at about 44 kDa (Figure 5D). Although this scheme is speculative, it could explain the pattern of the detected peptides.

A third hypothesis relies on the detection of another efficient translation initiation site on the ORF 2 sequence. The ATG codons at positions 869 and 920 of RNA 2 following the STOP codon (position 845) of ORF 3 are associated with a putative Kozak sequence (GxxATGT). These codons are in a suboptimal context. Nevertheless, an occurrence of a potential downstream secondary structure at positions 13–17 of ATG can enhanced the translation efficiency of some viral RNA [39,40] with this suboptimal context. Using the software, AUG_hairpin [29], the ATG codons at positions 869 and 920 possess a putative downstream hairpin, located at the distance of 15 nucleotides for 869 and 18 nucleotides for 920. Furthermore, no peptide of ORF 2 protein was identified in the part between the nucleotides 869 and 920 of RNA 2. These ATG codons could be candidate for the translation of a short ORF 2 (about 44 kDa) (Figure 5B).

According to the composition of pSP, the *N*-terminal region (residues 4 to 17) contains a high number of arginine residues and the peptide has a predicted α-helical secondary structure. Short regions containing basic amino acids (approximately 8–20 residues) are commonly used to bind RNA and can even recognise a specific RNA structure [41]. Arginine- and lysine-rich motifs (ARM and LRM) have been found in capsid proteins of several insect viruses, such as members of the genus *Alphanodavirus*, but also in plant viruses, such as members of the genera *Bromovirus*, *Dianthovirus* and *Tombusvirus*. For these genera of viruses, ARM deletions experiments [42,43] and substitutions of arginine or lysine residues [44,45] have demonstrated that this *N*-terminal region has a multifunctional nature. Indeed, ARM and LRM play a crucial role for RNA packaging and subsequent stable virion formation. In addition, this motif in *Bromovirus* and *Dianthovirus* is involved in infection and the cell-to-cell movement. Together with the proteomic results, the presence of positively charged sequences at the *N*-terminus of pSP reinforces the hypothesis that pSP is a structural protein of CBPV. In a recent study Kuchibhatla *et al.* [41] found some homologies between the RNA 2 ORF 3 encoded protein and membrane proteins from insect viruses, but they did not detect any homologs using HHpred. In the present study, HHpred analysis of pSP amino acids sequence revealed sequence similarity to the *N*-terminus part of the capsid protein alpha (39.6%) of Nodamura virus, a virus belonging to family *Nodaviridae* genus *Alphanodavirus*. However, due to low sequence complexity, this result does not imply that the proteins are homologous. Interestingly, the N-terminal region of capsid protein alpha of Nodamura virus contains ARM. Prior entry into cell, protein alpha undergoes cleavage yielding capsid proteins beta and gamma. In genus *Alphanodavirus*, capsid protein gamma is known to correspond to the hydrophobic C-terminal region of capsid protein alpha and is essential for breaching the host membrane, enabling capsid entry, as shown in some non-enveloped viruses [46]. Thus, when the viral particle is in contact with the cell, the hydrophobic region is exposed for membrane breaching. The CBPV virion may share features of protein conformation with these viruses. Furthermore, the hydrophobicity plot of fusion capsid protein (p65) revealed one hydrophobic *C*-terminal region corresponding to the *C*-terminal region of hSP and one cleaved protein of about 18 kDa. Therefore, this fragment protein could have the same role than the capsid protein gamma of family *Nodaviridae*. For CBPV, the interaction of ARM with RNA could facilitate the initiation of p65 cleavages leading to mature capsid proteins. Further work is needed to confirm these hypotheses.

In conclusion, this study demonstrates that the CBPV virion contains at least two proteins, which are encoded by ORF 2 and ORF 3 of RNA 2. The pattern of the peptides identified from RNA 2 ORF 3, suggest that CBPV encodes another capsid protein, which could result from the continuous translation of ORF 3 on ORF 2 or from a shorter ORF 2 translation product. Further work is needed to test these hypotheses. To this aim, given that naked CBPV RNAs are infectious [47,48] we are currently setting up a reverse genetics system as a tool to study the CBPV genome strategy.
